# RISE: A CONCEPTUAL MODEL OF INTEGRATED AND RESTORATIVE ELDER ABUSE AND SELF-NEGLECT INTERVENTION

**DOI:** 10.1093/geroni/igad104.1153

**Published:** 2023-12-21

**Authors:** David Burnes, M T Connolly, Erin Salvo, Patricia Kimball, Geoff Rogers, Stuart Lewis

**Affiliations:** University of Toronto, Toronto, Ontario, Canada; University of Southern California, Los Angeles, California, United States; Department of Health and Human Services, Augusta, Maine, United States; Elder Abuse Institute of Maine, Brunswick, Maine, United States; City University of New York, New York City, New York, United States; Dartmouth College, Lebanon, New Hampshire, United States

## Abstract

Despite a growing number of elder abuse and self-neglect (EASN) cases nationwide, community-based EASN response programs such as Adult Protective Services (APS) lack a conceptually driven, defined, prolonged intervention phase to address these complex situations. This presentation provides a conceptual overview and developmental description of RISE, a community-based model of EASN intervention that works alongside APS or other systems that interact with older adults who are at risk of or experiencing EASN. RISE was developed in consultation with APS caseworkers and supervisors, as well as practitioner and research stakeholders and experts from numerous sectors in Maine and nationwide, building bridges among varying stakeholders. Informed by ecological-systems, relational, and client-centered perspectives and adapting evidence-based or promising modalities from other fields (including motivational interviewing, restorative justice, teaming, supported decision making, goal attainment scaling, and engagement), the RISE model intervenes at levels of the individual older adult victim and others, including the alleged harmer, their relationship, and the systems of informal and formal support surrounding the victim-harmer dyad. The RISE model addresses an intervention gap in response systems to better meet the wishes and needs of EASN victims and others in their lives, leading to more sustainable outcomes. RISE has fostered new research partnerships between researchers and community advocates, who have become both research allies and contributors. RISE supports people with cognitive impairment to make their own decisions. RISE also empowers both older people and younger people in their lives, motivating change, and strengthening and restoring the relationships among them.

